# Neuromolecular Basis of Impaired Conditioned Taste Aversion Acquisition in Valproate-Induced Rat Model of Autism Spectrum Disorder

**DOI:** 10.3390/genes16020203

**Published:** 2025-02-07

**Authors:** Tapasya Pal, Savannah Harvey, Allen S. Levine, Pawel K. Olszewski, Anica Klockars

**Affiliations:** 1 School of Science, University of Waikato, Hamilton 3216, New Zealand; tapasya.pal@waikato.ac.nz (T.P.); pawel.olszewski@waikato.ac.nz (P.K.O.); 2 Department of Food Science and Nutrition, University of Minnesota, St. Paul, MN 55455, USA; aslevine@umn.edu

**Keywords:** conditioned taste aversion, autism, feeding behavior, aversive learning

## Abstract

Background/Objectives: Autism spectrum disorder (ASD), defined by social, behavioral, and cognitive anomalies, is also associated with dysregulated appetite. ASD individuals, often described as “picky eaters”, exhibit restricted dietary preferences and a pronounced avoidance of novel foods. This suggests that the perceived safety of specific tastants may be a crucial determinant of dietary acceptance in ASD. Here, we explore the hypothesis that conditioned taste aversion (CTA), a learned avoidance of foods whose intake promotes sickness, is exacerbated in ASD. Methods: We assessed the magnitude of a lithium chloride (LiCl)-induced CTA in the valproic acid (VPA) rat model of autism versus in healthy control rats. We also examined the effect of a standard 3 mEq LiCl dose on transcript and neuronal activation changes in brain circuits mediating feeding behavior and associative learning. Results: Surprisingly, we found that while 3 mEq LiCl induced CTA in healthy controls, even the 6 mEq dose was ineffective in generating aversion in VPA rats. LiCl at 3 mEq affected c-Fos immunoreactivity in the hypothalamus and amygdala in controls, whereas in VPA rats it did not produce any c-Fos changes. Gene expression analysis of feeding-related genes (AgRP, NPY, OXT) and those involved in regulating stress and anxiety (DOR and MC3R) were differentially regulated in the VPA rats. Interestingly, transcripts for COMT1, AgRP, OXT, and MC3R were downregulated in saline-treated VPA rats compared to saline-treated controls. Conclusions: We conclude that VPA rats show blunted CTA responsiveness, which is reflected by a differential impact of LiCl on circuits that promote the acquisition of CTA in healthy versus autistic individuals.

## 1. Introduction

Feeding satisfies an organism’s metabolic and nutritional needs but also carries the risk of ingesting toxic substances. Complex mechanisms, such as hyponeophagia and bitter taste avoidance, limit the consumption of potentially harmful substances [1,2,3]. One of these mechanisms, conditioned taste aversion (CTA), develops when the intake of a novel food is associated with gastrointestinal (GI) discomfort (in a laboratory setting, it is typically induced by a post-meal injection of a noxious substance, such as lithium chloride (LiCl) [4]). A subsequent presentation of this food affects a broad array of gustatory and stress-related neural circuits, leading to avoidance, hypophagia, and decreased preference for this tastant [4,5,6,7,8,9,10,11].

Autism spectrum disorder (ASD) is a neurodevelopmental disorder as defined by the Diagnostic and Statistical Manual of Mental Disorders (DSM-5) alongside attention deficit/hyperactivity disorder (ADHD) and intellectual disability (ID). There is a strong genetic component underlying the manifestation of ASD with numerous genes being implicated. Genetic variants within specific chromatin remodeling (CHD8) [12], ion channel function (SCN1A and SCN2A) [13], synaptic development and transmission (CDKL5, STXBP1 and STX1A) [14,15,16,17,18], transcriptional regulation (MECP2, FOXP1 and NAA15), as well as tumor suppressor (PTEN) genes include the most pathogenic variants associated with ASD [19,20,21,22]. Furthermore, genetic variants associated with ASD often co-occur with other neurodevelopmental disorders, such as ID [23,24]. ASD is most commonly discussed in the context of social behavioral and cognitive abnormalities and is also characterized by dysregulated appetite. Importantly, autistic individuals are described by their caregivers as ‘picky eaters’ with very narrow dietary preferences and excessive novelty- and other stressor-induced avoidance of foods, which has led to the speculation that the perceived safety associated with tastant intake is one of the most critical factors in diet acceptance in ASD [25,26,27,28]. The maladaptive behavior common in autism that has put particular focus on the formation of aversions in ASD is pica, the consumption of non-edible items including sand, chalk, clay, paper, or soap, seen as analogous to emesis [29,30,31]. Furthermore, aberrant Pavlovian learning responses have been reported in some transgenic animal models of ASD, though the neural bases of those remain unknown [32,33]. Finally, aberrant reward processing in ASD, already known to shape diet preferences in ASD individuals, may also be reflected through the abnormal processing of noxious stimuli [34,35].

In this study we utilized rats prenatally exposed to valproic acid (VPA). VPA is a short chain fatty acid which is commonly prescribed as an anti-epileptic drug [36]. Although VPA is an effective treatment for epileptic seizures, it has ASD-inducing teratogenic effects [37]. VPA rats exhibit disrupted histone acetylation, excitatory/inhibitory ratio neural circuit imbalances, abnormal neurogenesis, and altered neuronal organization and migration. ASD-associated behavioral phenotypes, such as lowered pain sensitivity, repetitive behavior, and increased latency for social interactions, are also observed [38]. Little research has investigated the acquisition of CTA in ASD [32,33], and, to the best of our knowledge, no research has investigated the underlying neuromolecular basis of this anomalous behavior. We employed c-Fos immunoreactivity and qRT-PCR to investigate the changes in neuronal activation and gene expression associated with the CTA response in ASD. We explored genes related to food intake (AgRP, NPY, OXT, OXTR, MC3R), opioid signaling (KOR, MOR, DOR), stress regulation (CRH), synaptic plasticity (PSD95, synapsin1), and catecholamine metabolism (COMTD1), molecular pathways known to be dysregulated in ASD [39,40,41,42,43]. 

In the current set of studies we have sought to investigate systematically whether the ASD phenotype involves excessive CTA responsiveness in VPA-rats. Specifically, we determined whether LiCl at doses known to produce a CTA in non-ASD rats generate CTA in VPA animals. Subsequently we conducted c-Fos immunoreactivity (IR) experiments in the broad network of sites that govern consummatory behavior in VPA versus control rats. Finally, we determined the effect of the LiCl injection on mRNA levels in the hypothalamic paraventricular nucleus (PVN) and central nucleus of the amygdala (CEA), two brain areas that play a key role in CTA acquisition.

## 2. Materials and Methods

### 2.1. Animals

Sprague-Dawley rats housed in standard Plexiglas cages with wire tops in a temperature-controlled (22 °C) animal room at an average humidity of 70% with a 12:12 light–dark cycle (lights on at 7:00 am) were provided with ad libitum access to standard laboratory chow pellets (Sharpes, Wellington, New Zealand; energy density: 3.6 kcal/g) and water unless otherwise specified. The animals were monitored daily for signs of illness or distress. The University of Waikato Animal Ethics Committee approved the procedures described herein (Protocol #1155).

### 2.2. Sodium Valproate Exposure

In utero VPA-exposed animal models were generated as described previously [40]. In sum, 12–16 weeks old adult female Sprague-Dawley rats were mated overnight with age-matched Sprague-Dawley males. Early the next day, vaginal smears were collected and stained with 1% crystal violet. Upon identification of spermatozoa, the animal was assigned as gestational day E0.5. Females received a single intraperitoneal (i.p.) injection of either 500 mg/kg sodium valproate (Sigma, Shanghai, China) or isovolumetric physiological saline (0.9% NaCl) i.p. on E12.5. Animals were weighed regularly, and female rats treated with sodium valproate were healthy.

Females were allowed to nurse and raise their offspring until weaning on postnatal day (PND) 25. Offspring prenatally exposed to physiological saline are referred to as controls, while those exposed to sodium valproate (VPA) are referred to as VPA. Only male offspring were used in the experiments.

**Figure 1:**
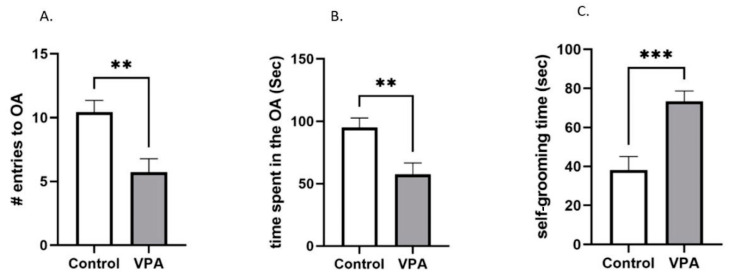
Phenotypic validation of ASD-like phenotypes using Elevated Plus Maze. (**A**) Number of entries to the open arm. VPA rats entered the open arms less often than controls. (**B**) Time spent in the open arm was recorded. VPA rats spent more time in closed arms. (**C**) Elevated self-grooming was observed in these animals. Data are expressed as mean ± SEM, N = 12–14/group. ** *p* < 0.01, *** *p* < 0.001.

The VPA rats were tested for the anxiety-like component of the ASD phenotype in the elevated plus maze test as described previously for the ASD behavioral confirmation [40]. The time spent and number of entries into the open arm were noted, along with the time spent in the closed arms for every rat. To assess whether the observation was merely a collateral effect of anxiety owing to a novel environment, self-grooming time was also evaluated as a parameter of anxiety-like behavior. Data were tested for normality and, upon confirmation of normal distribution, an independent two-sample Student’s *t*-test was performed. Values were considered significantly different for *p* ≤ 0.05. VPA rats displayed increased self-grooming (t(24) = −4.03903; *p* = 0.000477) in the maze. There was a reduced number of entries to the open arms (t(24) = 3.27469; *p* = 0.003204) coupled with a lesser amount of time spent in the open arms (t(24) = 3.12196, *p* = 0.004636) in these animals (Figure 1).

Age-matched VPA animals were lighter than their healthy controls (control 584.625 ± 9.377511741; VPA 461.5 ± 20.11315214) (t(32) = 6.349; *p* < 0.00001) as we have reported earlier [40].

### 2.3. Establishing Responsiveness to i.p. LiCl

Adult VPA and their age-matched control animals were randomly assigned to groups ensuring no difference in body weight between groups. VPA animals weighed slightly less than the controls as we reported previously [40].

Experimentally novel adult control animals deprived of water overnight were given 0.1% saccharin solution for 1 h. After 1 h, saline or LiCl (0.6 mEq/kg, 3 mEq/kg or 6 mEq/kg) (N = 8/group) were administered i.p. Forty eight hours later, the animals were deprived of water overnight (16 h). Water and 0.1% saccharin were then presented simultaneously and the 2-h intake was measured.

In order to establish the lowest effective dose of LiCl in each phenotype (autistic VPAs and non-autistic controls), data from LiCl-injected groups were compared with the saline group using one-way ANOVA for each phenotype, followed by Dunnett’s post-hoc test separately for control and VPA animals. No data normalization was applied prior to statistical analysis. Values are presented as means ± SEM and were considered significantly different when *p* ≤ 0.05.

### 2.4. Effect of LiCl on c-Fos Immunoreactivity in the Brain in VPA Versus Control Rats

Immunohistochemistry was performed by utilizing the previously described protocol [40]. Saline or the lowest effective dose of LiCl (3 mEq/kg) (N = 8–10 per group) was administered intraperitoneally to the adult control and VPA animals. Sixty min after the administration, animals were deeply anesthetized with 35% urethane dissolved in 0.9% saline. After confirming the absence of toe-pinch and palpebral reflexes, transcardial perfusion was conducted using 50 mL of saline, followed by 500 mL of 4% paraformaldehyde in phosphate-buffered saline (pH 7.4). The brains were then extracted, sectioned coronally at 60 μm with a vibratome (Leica, Wetzlar, Germany), and prepared as free-floating sections for immunostaining.

Sections were rinsed in Tris-buffered saline (TBS, pH 7.4–7.6), and then incubated for 10 min in 3% H_2_O_2_, 10% methanol (diluted in TBS) at room temperature. After rinsing in TBS, they were incubated at 4 °C for 18 h in rabbit-anti-Fos monoclonal primary antibody (diluted 1:12,000; Synaptic Systems, Sydney, Australia). Sections were washed in TBS and incubated for 1 h at room temperature in biotinylated-secondary goat-anti-rabbit antibody (1:400; Vector Laboratories, Burlingame, CA, USA). Following four washes in TBS, sections were incubated for 1 h with avidin–biotin peroxidase complex (1:800; Elite Kit, Vector Laboratories). All antibodies were dissolved in a solution of 0.25% gelatin and 0.5% Triton X-100 in TBS. The staining in the tissues was visualized with 0.05% diaminobenzidine (DAB, Sigma), 0.01% H_2_O_2_ and 0.3% nickel sulfate (15–20-min incubation). Sections were washed thoroughly in TBS, mounted onto gelatin-coated slides, air-dried overnight, dehydrated in ascending concentrations of ethanol followed by xylene, and embedded in Entellan (Merck KGaA, Darmstadt, Germany).

Brightfield images of immunohistochemically stained brain sections were acquired using an OMAX digital microscope camera attached to a Nikon Eclipse 400 microscope (Auckland, New Zealand). Images were not processed digitally before or after the analysis; images across all groups were captured with the same brightness, contrast, resolution, and aspect ratio. The number of c-Fos-positive nuclei per mm^2^ was counted bilaterally for each neuroanatomical region of interest using ImageJ (v. 1.54h; Fiji), with boundaries defined according to the Paxinos and Watson brain atlas. Thus, the comparison of the activation levels reflects the density rather than the raw number of Fos-positive neurons.

### 2.5. Gene Expression Analyses

#### 2.5.1. Experimental Groups and Microdissection

Control and ASD animals were divided into two groups each (n = 10 animals/group). Animals received an i.p. injection of either vehicle (0.9% saline) or 3 mEq/kg LiCl (food was removed just prior to the injection). One hundred min later, animals were decapitated, brains were immediately excised, and microdissection of the PVN and CEA was carried out. Tissues were immersed in RNAlater (Ambion, Thermo Fisher Scientific, Auckland, New Zealand) at room temperature for 2 h before freezing at −80 °C until further processing.

#### 2.5.2. qRT-PCR Protocol and Data Analysis

Primers were selected based on the literature review and in silico validation using Primer-BLAST (NCBI) and Multiple Primer Analyzer (Thermofisher, Scoresby, Australia). Previously validated primers were prioritized, and new primers were designed to span exon–exon junctions, with optimal melting temperatures (~60 °C) and amplicon sizes (100–200 bp). Primer specificity was confirmed using BLAST. A standard method of qPCR sample preparation was employed, as previously described [44]. Tissues stored in RNAlater were thawed on ice and homogenized in 100 µL Trizol (Ambion, Glasgow, UK). Twenty µL chloroform was added before centrifugation at room temperature for 10 min at 10,000× *g*. The clear phase containing total RNA was isolated and precipitated using 0.5 mL cold isopropanol and was incubated in an ice bath for 10 min before another round of centrifugation at 4 °C for 10 min at 10,000× *g*. The supernatant was discarded carefully, keeping the pellet intact before 300 µL of 75% ethanol prepared in DEPC-treated water was added, and the pellet was washed via centrifuging at 4 °C for 10 min at 10,000× *g*. The supernatant was again removed, and the pellets were air-dried.

Dry pellets were dissolved in 8 µL DEPC water and 1 µL 10 × DNAse buffer (dNature, Gisborne, New Zealand). Samples were incubated with 1 µL DNAse (dNature) for 30 min at 37 °C. The reaction was then halted using 1 µL stop buffer before incubation for a further 10 min at 65 °C. Concentrations and the purity of RNA were measured (µg/µL) with a spectrophotometer.

cDNA was synthesized from RNA samples using an iScript Advanced cDNA synthesis kit (BioRad, Hercules, CA, USA). The quantification and purity of cDNA were determined using a nanodrop. Quantitative real-time PCR reactions were carried out in duplicate using 4 µL of 25 ng/µL cDNA, 1 µL of forward and reverse primers (5 µM) specific to the transcript (Table 1), 10 µL iTaw Universal SYBR Green Supermix (BioRad), and 4 µL MQ H_2_O. Expression of housekeeping genes (Actin b, TBP) were used to analyze normalization factors. Nuclease-free water was used as the template for negative controls for each transcript. NCBI-BLAST^®^ was used to check the specificity of primer pairs prior to use in reactions.

The amplification protocol used is as follows: denaturation at 95 °C for 15 min, followed by 45 cycles of 15 s at 95 °C, 15 s at the primer-specific annealing temperature, and 30 s at 72 °C. The final extension was at 72 °C for 30 s.

Thermal profiles of the amplified transcripts were visualized in CFX maestro using melt peaks, where Tm analysis of the negative value of the change in relative fluorescence units (RFU) over the change in temperature (°C) was plotted (−dRFU/dT) to determine the specificity of the primers to a given transcript and primer dimer.

### 2.6. Data Analysis

Data from the LiCl dose response were analyzed using GraphPad Prism one-way ANOVA, followed by Dunnett’s post-hoc test for the control and VPA animals after validation of normal distribution using Shapiro Wilk, and the Kolmogorov Smirnov test for normality. Values were considered significantly different for *p* ≤ 0.05.

Densities of c-Fos-positive nuclear profiles (per mm^2^) were averaged per individual, and then per group. Data between the groups (control saline, control LiCl, and VPA saline, VPA LiCl) were compared using Two-Way ANOVA after performing the normality test using Shapiro Wilk and the Kolmogorov Smirnov test. Interaction between two factors (LiCl treatment and VPA exposure) were assessed and values were considered significantly different for *p* ≤ 0.05.

qRT-PCR data were analyzed using BioRad CFX Maestro software (v. 2.3; BioRad); rtPCR results were normalized with housekeeping genes (Actin B and TBP). The distribution of ΔCq values was checked using a Shapiro Wilk test for normality. ΔCq values were analysed with a two-way ANOVA assessing the interaction between LiCl treatment X VPA exposure. Values were considered significantly different when *p* ≤ 0.05.

The groups were non-blinded, and the authors were aware of the group allocations. No data points were excluded. Data are available upon request from the corresponding author.

## 3. Results

### 3.1. VPA Animals Are Resistant to CTA Acquisition

**Figure 2:**
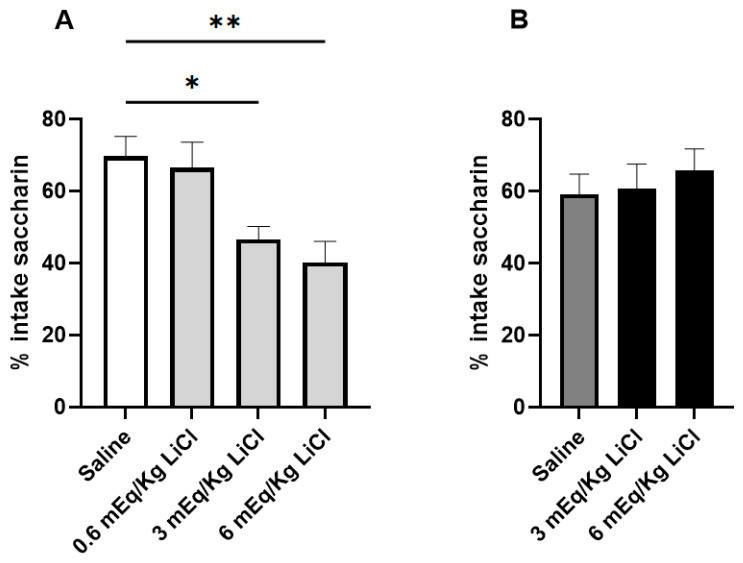
Effect of i.p. saline and LiCl on the acquisition of CTA to 0.1% saccharin. The graphs show the % intake of the saccharin solution during a two-bottle test in which a choice between water and saccharin was given. (**A**) Intake in control animals (N = 10/group). (**B**) Intake in VPA animals (N = 8/group). Data are expressed as mean ± SEM * *p* < 0.05, ** *p* < 0.01.

When examining the susceptibility to a CTA using a two-bottle test, the non-VPA control animals that had been treated with 3 mEq/kg and 6 mEq/kg LiCl ingested significantly less of the 0.1% saccharin solution than their saline-treated counterparts (F (DFn, DFd): F (3, 33) = 6.592; *p* = 0.001 adjusted P_3mEq/kg_ LiCl = 0.025; adjusted P_6mEq/kg_ LiCl = 0.002). On the other hand, the VPA animals did not develop a CTA, even at a dose as high as 6 mEq/kg LiCl (Figure 2).

### 3.2. i.p. 3 mEq/kg LiCl Fails to Drive Changes in c-Fos Immunoreactivity in the VPA Rats

**Figure 3:**
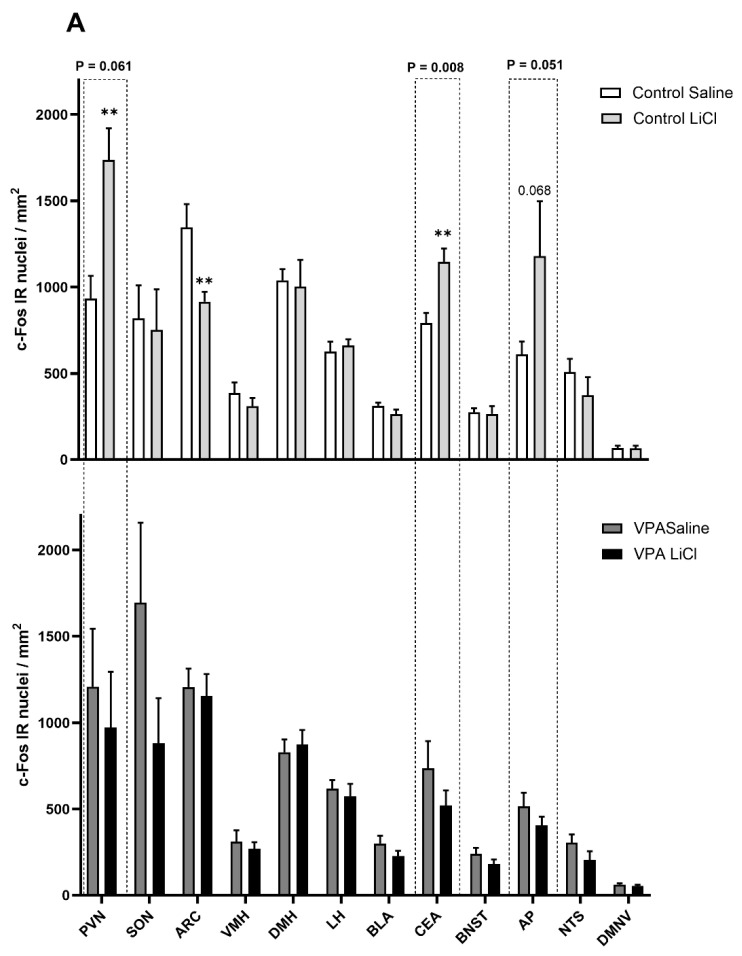

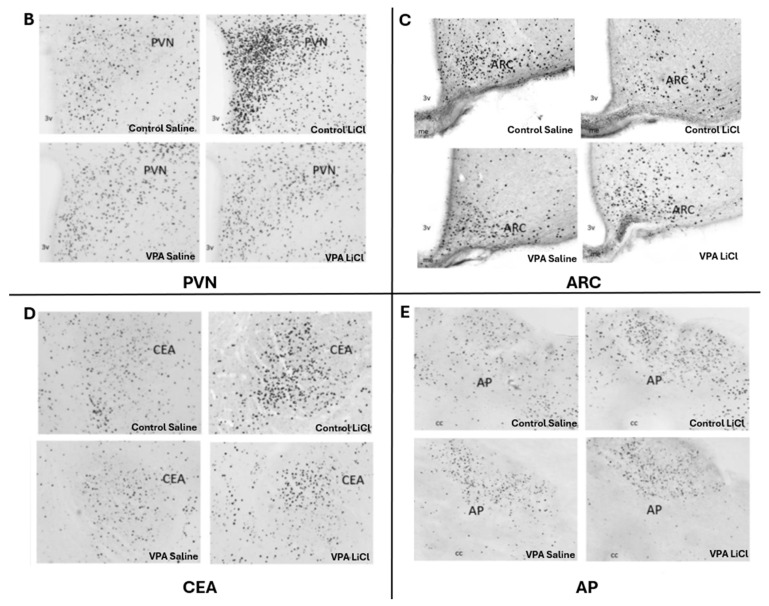
(**A**) c-Fos immunoreactivity in control and VPA animals injected i.p. with saline vs. 3 mEq/kg LiCl showing interaction between LiCl treatment and VPA exposure in the CEA and a trend towards elevated c-Fos in the PVN and AP. Representative photomicrographs depicting (**B**) PVN (**C**) ARC (**D**) CEA, and (**E**) AP with differential c-Fos levels in control animals after LiCl treatment. ** Significantly different from the control saline group. Boxes with *p* values above them indicate a significant LiCl treatment X VPA exposure interaction. AP, area postrema; ARC, arcuate nucleus; BLA, basolateral amygdala; BNST, bed nucleus of stria terminalis; CEA, central amygdala; DMH, dorsomedial hypothalamic nucleus; DMNV, dorsal motor nucleus of the vagus; LH, lateral hypothalamus; NTS, nucleus tractus solitarius; PVN, paraventricular nucleus of the hypothalamus; SON, supraoptic nucleus; VMH, ventromedial hypothalamic nucleus.

In the follow-up immunohistochemical analysis, we found differences in neuronal activation after saline or 3 mEq/kg LiCl in the VPA versus control rats. The control animals injected with LiCl showed significantly elevated c-Fos IR in the PVN and CEA and a trend in the AP, whereas c-Fos IR was significantly lower in the ARC (Figure 3A). In contrast, LiCl did not affect c-Fos IR in those regions in the VPA animals (Figure 3B). Two-way ANOVA shows an LiCl treatment X VPA exposure interaction in the CEA (F (1, 22) = 8.419; *p* = 0.008) and a trend toward interaction in the PVN (F (1, 27) = 3.815; *p* = 0.061) and in the AP (F (1, 25) = 4.192; *p* = 0.051) (Figure 3).

### 3.3. LiCl Injection Affects Expression of Different Transcripts in the VPA Rats than in Controls

**Figure 4:**
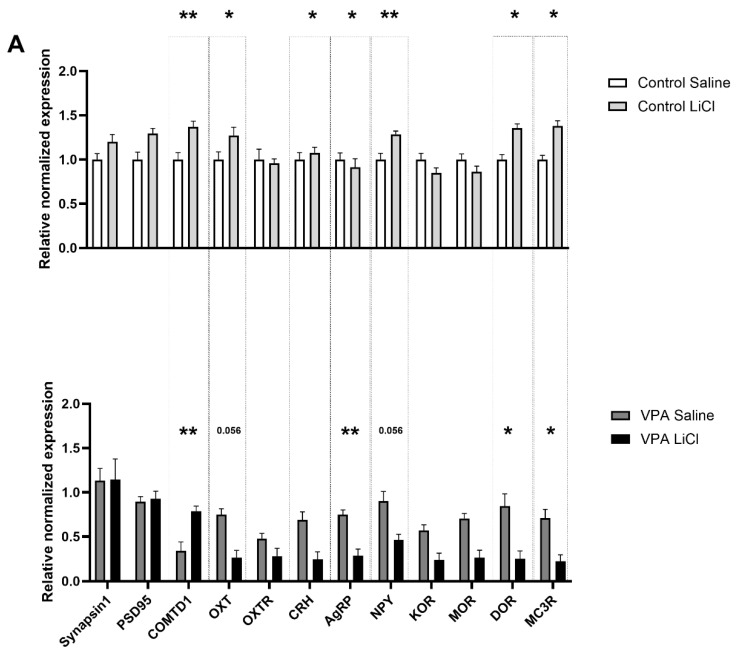

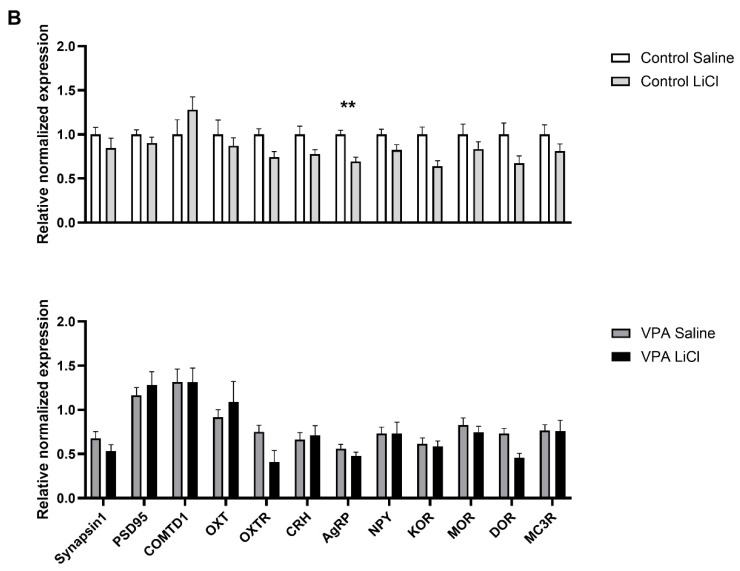
LiCl -induced mRNA level changes in the (**A**) CEA and (**B**) PVN of healthy control versus ASD VPA rats. PSD95—discs large MAGUK scaffold protein 4 (Dlg4), COMTD1—catechol-O-methyltransferase domain containing 1, OXT—oxytocin/neurophysin I prepropeptide, OXTR—oxytocin receptor, CRH—corticotropin releasing hormone, AgRP—agouti related neuropeptide, NPY—neuropeptide Y, KOR—opioid receptor kappa 1 (OPRK1), MOR—opioid receptor mu 1 (OPRM1), DOR—opioid receptor delta 1 (OPRD1), MC3R—melanocortin 3 receptor. * *p* < 0.05, ** *p* < 0.01.

In our follow-up gene expression analysis, we found differentially regulated genes in the CEA of the VPA rats after LiCl treatment. A two-way ANOVA for the gene expression analysis in the CEA showed a significant LiCl treatment and VPA exposure interaction for COMTD1 (*p* = 0.004), OXT (*p* = 0.013), CRH (*p* = 0.042), AGRP (*p* = 0.025), NPY (*p* = 0.003), DOR (*p* = 0.020), and MC3R (*p* = 0.012) (Table 2). Upon LiCl treatment, we observed upregulated COMTD1 (*p* = 0.004) in the VPA group while OXT (*p* = 0.056), AgRP (*p* = 0.007), NPY (*p* < 0.001), DOR (*p* = 0.027) and MC3R (*p* = 0.019) were significantly downregulated (Figure 4A). However, control animals displayed minimal changes in gene expression patterns in the CEA, except for DOR, which showed a trend towards upregulation (*p* = 0.063) (Figure 4A).

On the other hand, no significant LiCl treatment X VPA exposure interaction was noticed in the PVN. AgRP (*p* = 0.008) was significantly downregulated in the control group upon LiCl administration (Figure 4B).

## 4. Discussion

One of the most recognized facets of disordered eating behavior in ASD revolves around extreme pickiness in selecting food items. The refusal to eat appears to go far beyond typical taste and texture preferences, and it incorporates additional characteristics of a food item, such as color, and the precise manner and timing of presentation [25,26,45]. ASD individuals have a great difficulty in accepting food that is novel or whose presentation is associated with any aspects of unpredictability [46,47]. This has led to a speculation that food avoidance stemming from a perceived ‘lack of safety’ of a food item (familiar taste, presentation, and context of a meal) is exacerbated in ASD. Considering the above, one could easily presume that ASD individuals might also display excessive avoidance of foods whose consumption has led to unpleasant consequences, such as malaise. Surprisingly, our study utilizing a VPA ASD rat model, shows for the first time that a conditioned taste aversion is in fact suppressed in ASD and this effect is associated with a blunted c-Fos IR and transcript changes in the brain circuit that regulates consumption.

Consistent with previous studies, both control and VPA rats treated with saline following overnight water deprivation showed a preference for saccharin over water, consuming 60–70% saccharin [34,48]. LiCl-treated non-VPA rats exhibited aversion to saccharin, following treatment with a standard dose of LiCl (3 mEq/kg) as well as a high, 6-mEq dose. In those animals, water was slightly preferred over the sweet solution. On the other hand, LiCl-injected VPA animals failed to develop an aversion, even at a dose as high as 6 mEq/kg.

Altered structural and functional connectivity of the amygdala is well-established in ASD [49,50,51,52]. Electrolytic lesions of the CEA led to attenuated CTA in laboratory animals [53,54], highlighting the critical role of the CEA in CTA acquisition. LiCl treatment has been shown to drive elevated c-Fos IR in the CEA in rats [55,56,57,58,59,60,61,62,63,64,65]. Our data, showing elevated c-Fos IR in the CEA of healthy control rats, further corroborate these findings. However, in the VPA cohort, we observed no aversion-associated change in c-Fos IR in the CEA, suggesting a potential disruption to the amygdala’s involvement in the aversion response in autism. Our follow-up gene expression analysis of microdissected CEA revealed that LiCl injection affected the expression of a distinct set of transcripts in the VPA rats compared to the controls. Notably, feeding-related genes (AgRP, NPY, OXT [66]) and those involved in regulating stress and anxiety (DOR [67] and MC3R [68]) were differentially regulated in the VPA rats. One can speculate that a decrease might translate to a decreased anxiogenic effect of encountering a toxin which produces an unpleasant gastrointestinal sensation. A decreased anxiogenic component of LiCl treatment may hinder the process of acquiring aversion learning. Interestingly, transcripts for COMT1, AgRP, OXT, and MC3R were downregulated in saline-treated VPA rats compared to saline-treated controls. While it remains elusive whether this difference in gene expression in saline-treated VPA rats reflects their resistance to aversion acquisition compared to controls, or whether the differential gene expression underpins the blunted aversion response, it is evident that the neural circuits in ASD animals are differentially affected by LiCl treatment.

The hypothalamic PVN is a major site of neurogenic and humoral control of feeding behavior. LiCl treatment drives c-Fos IR in the hypothalamic PVN in rats [69,70,71,72]. In line with these earlier findings, our data also show an increase in the density of c-Fos positive nuclei in this area in the healthy control rats, but not in VPA animals. This suggests that the neurogenic and humoral pathways involved in feeding and stress regulation are disrupted in the VPA animals, indicating altered hypothalamic function in the context of autism. Our data showing downregulated AgRP in control animals indicate aversion-induced anorexia. The lack of change in gene expression regulating feeding [34,40] and stress response [73] might be the reason for the anomalous CTA acquisition in the VPA rats. Furthermore, VPA exposure disrupts critical developmental processes, such as neurogenesis, synaptogenesis, and neurotransmitter regulation, which could contribute to baseline gene expression differences [42,43] between the control and VPA groups.

The neuroanatomical positioning of the ARC enables it to sense various nutrients and hormones and convey neural information to other brain areas. The reduced c-Fos IR in the ARC thus possibly reflects the aversion-derived anorexia [74].

Peripheral (i.p.) administration of LiCl has been shown to activate the chemosensitive neurons in the AP [69], and a lesion in the AP causes attenuated CTA acquisition [59]. AP receives peripheral information through humoral routes as well as through splanchnic and vagal inputs. Adachi et al. demonstrated that some AP neurons responding to direct application of isotonic LiCl suggests that induction of CTA is possible even without the activation of the vagal inputs [75]. This is in line with our finding that, although there was an elevated number of c-Fos positive nuclei in the AP in the control animals, there was no difference in the DMNV upon exposure to LiCl. The lack of activation of the AP in the VPA animals may indicate altered chemosensation and/or an anomalous vagal afferent activity associated with ASD.

Kosaki et al. reported that BTBR mice, a neuroanatomical model of ASD, showed weaker latent inhibition of CTA [32]. In line with this, Strekalova and colleagues reported sialyltransferase knockout (St3gal5−/−) mice exhibiting ASD-like behaviors showed impaired CTA. Their report shows LiCl-treated mutants exhibiting no significant difference in sucrose preference during recall compared to saline-treated controls [33]. Additionally, ASD individuals often present pica, when non-edible substances are consumed, despite prior experiences of adverse physiological effects. Taken together, these indicate an impairment of associative learning in autism. Instead of a generalized aversion, individuals with ASD seem to lack the context-specific inhibitory learning mechanisms that would typically protect against the consumption of harmful substances. This deficit may explain the persistent underfeeding we observed previously [40], which likely serves as a compensatory protective response to mitigate the risk of ingesting toxic agents, highlighting the adaptive challenges in aversive learning in ASD.

## 5. Conclusions

In sum, our findings demonstrate a lack of CTA acquisition in ASD VPA animals even after 6 mEq/kg LiCl administration, in contrast to healthy controls which showed expected aversive responses at 3 mEq/kg. This, combined with differential brain activity and gene expression changes in the VPA animals reveal the neuromolecular basis of disrupted associative learning. Our gene expression analyses indicate atypical regulation of genes involved in feeding, learning, and fear generalization. These data provide evidence indicating impaired context-specific inhibitory learning, such as CTA, in autism.

One should note that the current study provides only a small subset of genes involved in aversive responsiveness. While being informative in that, indeed, VPA animals do not exhibit a proper molecular response to the LiCl challenge, a much broader transcriptomic analysis is necessary in order to better understand the breadth of changes and potential therapeutic targets to ameliorate the issue of dampened CTA learning in ASD individuals. Furthermore, one should note that CTA learning and learning processes in general are age-dependent. Therefore, future studies should also include additional age groups in order to ascertain whether aberrant CTA processing is a lifelong phenomenon in ASD or whether its occurrence is restricted to select life stages. 

## Figures and Tables

**Table 1:** List of all primers used in qRT-PCR experiments. PSD95—discs large MAGUK scaffold protein 4 (Dlg4), COMTD1—catechol-O-methyltransferase domain containing 1, OXT—oxytocin/neurophysin I prepropeptide, OXTR—oxytocin receptor, CRH—corticotropin releasing hormone, AgRP—agouti related neuropeptide, NPY—neuropeptide Y, KOR—opioid receptor kappa 1 (OPRK1), MOR—opioid receptor mu 1 (OPRM1), DOR—opioid receptor delta 1 (OPRD1), MC3R—melanocortin 3 receptor.

Housekeeping Genes		
**Gene**	**Forward**	**Reverse**
*Actin b*	5′-AGTGTGACGTTGACATCC GT-3′	5′-TGCTAGGAGCCAGAGCAGTA-3′
*TBP*	5′-AGAACAATCCAGACTAGCAGA-3′	5′-GGGAACTTCACATCACAGCTC-3′
**Genes of Interest**		
**Gene**	**Forward**	**Reverse**
*MC3R*	5′-AGCAACCGGAGTGGCAGT-3′	5′-GGCCACGATCAAGGAGAG-3′
*AgRP*	5′-CAGAGTTCTCAGGTCTAAGTC-3′	5′-TTGAAGAAGCGGCAGTAGCAC-3′
*NPY*	5′-AGGTAACAAACGAATGGGGCT-3′	5′-TGATGTAGTGTCGCAGAGCG-3′
*COMTD1*	5′-TGTGTGCGGAACCTAAACGA-3′	5′-GAAGGTCGCGTGTTCCAGTA-3′
*CRH*	5′-TGGATCTCACCTTCCACCTT-3′	5′-TTCATTTCCCGATAATCTCCA-3′
*MOR*	5′-CGGACTCGGTAGGCTGTAAC-3′	5′-CCTGCCGCTCTTCTCTGG-3′
*KOR*	5′-AGACCGCAACCAACATCTACAT-3′	5′-GCACAGAACATCTCCAAAAGG-3′
*DOR*	5′-GCRACATTGCGGTCTGCCAC-3′	5′-CGAAGGCGAAGAGGAACACG-3′
*OXT*	5′-GACGGTGGATCTCGGACTGAA-3′	5′-CGCCCCTAAAGGTATCATCACAAA-3′
*OXTR*	5′-GATCACGCTCGCCGTCTA-3′	5′-CCGTCTTGAGTCGCAGATTC-3′
*Synapsin1*	5′-CACCAGGATGAAGACAAGCA-3′	5′-GTCGTTGTTGAGCAGGAGGT-3′
*PSD95*	5′-CTTCTCAGCCATCGTAGAGG-3′	5′-GAGAGGTCTTCAATGACACG-3′

**Table 2:** Two-way ANOVA gene expression statistics summary for the LiCl treatment X VPA exposure interaction.

Gene	F (DFn, DFd)	*p* Value
COMTD1	F (1, 33) = 9.304	*p* = 0.004
OXT	F (1,31) = 6.904	*p* = 0.013
CRH	F (1, 33) = 4.496	*p* = 0.042
AgRP	F (1, 30) = 5.544	*p* = 0.025
NPY	F (1, 34) = 9.861	*p* = 0.003
DOR	F (1, 31) = 5.996	*p* = 0.020
MC3R	F (1, 32) = 7.068	*p* = 0.012

## Data Availability

The data presented in this study are available upon reasonable request from the corresponding author.
